# Qualitative evaluation of selected social factors that impact sexual risk-taking behaviour among African students in Kwazulu-Natal, South Africa

**DOI:** 10.1080/17290376.2016.1218792

**Published:** 2016-08-17

**Authors:** Ndumiso Daluxolo Ngidi, Sibusiso Moyo, Thobile Zulu, Jamila Khatoon Adam, Suresh Babu Naidu Krishna

**Affiliations:** ^a^ Master’s Degree in Development Studies, PhD Candidate, is a Projects Officer at the Department of Counselling and Health, Durban University of Technology, Durban 4000, South Africa; ^b^ PhD in Mathematics, Masters in Tertiary Education Management, is a Acting DVC Engagement and Director at the Research and Postgrad Support at the Durban University of Technology, Durban 4000, South Africa; ^c^ Masters Degree in Social Sciences, is a HIV/AIDS Center Manager at the Department of Counselling and Health, Durban University of Technology, Durban 4000, South Africa; ^d^ D Tech in Clinical Technology, is a Professor at the Department of Biomedical & Clinical Technology, Faculty of Health Sciences at the Durban University of Technology, Durban 4000, South Africa; ^e^ PhD, is a Research Associate at the Department of Biomedical & Clinical Technology, Faculty of Health Sciencesat the Durban University of Technology, Durban 4000, South Africa

**Keywords:** HIV/AIDS, prevention, risky sexual behaviour, sexual relationships, university students, VIH/SIDA, prévention, comportement sexuel à risque, relation sexuelle, étudiants

## Abstract

Background: The incidence of HIV and AIDS continues to be a source of great concern within universities in South Africa. Furthermore, university students constitute an important community in the intervention against the HIV/AIDS epidemic. Students in the age group of 15–24 years are at a greater risk of HIV infection than any other group in the country; yet, little is known about why they continue to engage in risky sexual practices. Objectives: This study was designed to explore the sexual behaviour of students in a metropolitan Durban University of Technology in KwaZulu-Natal to understand the social factors underlying their risk of HIV infection. Methods: This is a qualitative study that used cluster sampling where the population was stratified by campus and faculty. The study population was selected using a standard randomization technique. This was a part of a multi-phased research project aimed at providing a sero-prevalence baseline and an analysis of risk-taking behaviour at a Durban University of Technology in the eThekwini Metropolitan Municipality area. Results: The study highlights peer pressure among students as an influence in promoting high-risk sexual behaviour. Within this context, the findings revealed that university students lack the ability to negotiate risk-aware decisions especially regarding sexual relationships. Conclusion: This study draws attention to the perspectives of African university students regarding their risk-taking sexual practices and selected factors which influence such behaviour. The findings are not exhaustive in exploring contextual antecedents that shape students’ sexual practices. However, they provide an important basis in understanding key factors which expose students to HIV infections. The study provides insights into opportunities for further studies as well as preventative implications.

## Résumé

Historique: Le taux du VIH et du SIDA demeure une source de préoccupation majeure au sein des universités sud-africaines. En outre, les étudiants sont les premiers concernés par la lutte contre le VIH/SIDA. Les 15–24 ans sont les plus vulnérables au VIH dans le pays. Or les raisons expliquant l'adoption des pratiques sexuelles risquées ne sont toujours pas connues. Objectifs: Cette étude, menée au sein d'une université technique du KwaZulu-Natal, vise à explorer le comportement sexuel des étudiants afin de comprendre les facteurs sociaux sous-jacents liés aux risques d'infection par le VIH. Méthodes: Il s’agit d'une étude qualitative utilisant l'échantillonnage en grappes dans les cas où la population est stratifiée par campus ou faculté. Les personnes sondées étaient sélectionnées par une technique de randomisation standard. Ce projet de recherche se déroulera en plusieurs étapes. Il vise à fournir une base de référence ayant une prévalence nulle, et une analyse des pratiques à risque à Durban University of Technology (Université technique de Durban) située au sein de la municipalité métropolitaine d’eThekwini. Résultats: Cette étude met en exergue le lien entre la pression sociale la persistance des comportements sexuels à risque. Dans ce contexte, les conclusions de cette étude indiquent que les étudiants sont incapables de prendre des décisions réfléchies en ce qui concernent les pratiques à risque, notamment leurs relations sexuelles. Conclusion: Cette étude met en relief les impressions des étudiants africains sur leur comportement sexuel à risque et l'influence exercée par des facteurs sociaux sur ce dernier. Les résultats de cette enquête ne représentent pas de manière exhaustive le rôle du milieu contextuel sur le développement des pratiques sexuelles des étudiants. Or, ils posent une base importante pour comprendre des facteurs clés qui exposent les étudiants à l'infection VIH. Cette étude pourrait servir de point de départ à des recherches plus avancées, ainsi qu'à des projets de prévention.

## Introduction

The incidence of HIV and AIDS continues to be a source of great concern within universities in South Africa (HEAIDS 2010). Students, who also fall within the age range of high national HIV prevalence, are at a greater risk of an HIV infection than any other group in the country (Maharaj & Cleland 2011; Mutinta, Govender, Gow & George 2012). Factors associated with student life such as sexual experimentation, inconsistent condom use, alcohol and drug use, and sex with multiple partners render this group of young adults susceptible to HIV (Abels & Blignaut 2011; Mutinta, Govender, Gow & George 2013). It is estimated that more than 300,000 university students in southern Africa are infected with HIV, and that 15% will experience unwanted pregnancies during their studies (WHO 2007) while an estimated 60% of unplanned pregnancies around the world occur among university students (UNAIDS 2013).

There is an abundance of literature in South Africa that has discussed sexual behaviour among young people, a group that university students generally fall within. However, these enquiries have largely focused on young people in rural KwaZulu-Natal (KZN) (Harrison, Xaba, Kunene & Ntuli 2001; Kharsany, Buthelezi, Frohlich, Yende-Zuma, Samsunder, Mahlase, *et al*. 2014), secondary schools (Bhana & Pattman 2009; Campbell & MacPhail 2002; James, Reddy, Taylor & Jinabhai 2004; Karim, Kharsany, Leask, Ntombela, Humphries, Frohlich, *et al*. 2014) and specific youths in townships (MacPhail & Campbell 2001; Pettifor, Lippman, Selin, Peacock, Gottert, Maman, *et al*. 2015). Other studies have explored young people’s sexuality through their parents’ perspective (Madumo, Havenga & Van Aswegen 2015; Rau, Radloff, Coetzee, Nardi, Smit & Matebesi 2015). Likewise, studies in this field of enquiry among students have generally employed a quantitative approach (Blignaut, Jacobs & Vergnani 2015). Furthermore, majority of available literature predominantly focuses on youth risky sexual practices (Simbayi, Shisana, Rehle, Onoya, Jooste, Zungu, *et al*. 2014).

On the contrary, qualitative enquiries among university students have investigated condom use (Pletzer 2000), sexual lifestyles in relation to ethnicity (Eaton, Flisher & Aarø 2003), attitudes towards HIV testing (Peltzer, Nzewi & Mohan 2004) and transactional sex (Leclerc-Madlala 2003) and are a bit outdated. Furthermore, there is relatively little research focusing on the factors that influence sexual risk-taking behaviour in urban set-up among students and besides the majority of literature available focuses on rural KZN. This study was directed as a part of a multi-phased research project aimed at providing a sero-prevalence baseline and an analysis of risk-taking behaviour at Durban University of Technology (DUT) in the eThekwini Metropolitan Municipality area of KZN. Furthermore, this study as well seeks to address African student’s perspectives about factors that shape their sexual practices, thus rendering them vulnerable to HIV infection by exploring factors that influence sexual risk-taking behaviour among university students with prominence particularly to female students in DUT.

## Sexual risk, inexperience and vulnerability of students

In South Africa, the country with the world’s largest HIV-positive population, HIV infection rates are eight times higher among women in the teenage years than among young men of the same age (Harrison, Colvin, Kuo, Swartz & Lurie 2015; Simbayi, Shisana, Rehle, Onoya, Jooste, Zungu, *et al*. 2014), a pattern that has remained unchanged for over a decade. In the same age group, both earlier and recent studies have found that HIV disproportionately affects females, Africans, and those who reside in KZN (Graham & Mphaphuli 2015). The South African National HIV Prevalence, Incidence, Behaviour and Communication Survey of 2012 estimated that amongst individuals in the 15–49-year age group, the overall HIV prevalence was 18.8% [95% Confidence interval (CI) 17.5–20.3]. However, HIV is unevenly distributed across all provinces and the worst-affected province is KZN with a prevalence of 27.9% (95% CI 25.2–30.8) compared to the Western Cape which has a prevalence of 7.8% (95% CI 5.5–10.9) (Shisana, Rehle, Simbayi, Zuma, Jooste, Zungu, *et al*. 2012). Also, among young Africans, prevalence was estimated at 8% when compared to 0.3% for the White youths, 1.1% for the Coloured youths, and 0.8% for the Indian youths.

Within tertiary institutions in the country, the estimated HIV prevalence in 2009 was 3% among students. Within this HIV-positive group, 4% reported having had sex, while 7% of men and 12% of women reported symptoms of a sexually transmitted infection (STI) (HEAIDS 2010). Female students were reported as being three times more likely to be HIV positive when compared to their male counterparts. Moreover, HIV prevalence in the 18- to 19-year age group was 0.1%, a relatively lower percentage when compared to students in the 20–25-year groups (2%).

Despite a number of interventions aimed at addressing HIV infections within universities, risk-taking sexual practices continue to prevail and pose a serious health concern (Heeren, Jemmott III, Ngwane, Mandeya & Tyler 2013). The Higher Education HIV and AIDS Programme (HEAIDS) reports that in South Africa, only 62% of sexually active students had used a condom in their last sexual intercourse, 23% had sex while under the influence of alcohol, and only 48% had ever tested for HIV. The latter attests to the argument which suggests that university students in South Africa have negative attitudes towards HIV testing (Peltzer 2005; Peltzer *et al*. 2004). This renders them more vulnerable to infection and poses a serious risk for their sexual partners. There is a dearth of literature that explores why university students continue to engage in risky sexual practices.

Some researchers have argued that peer education programmes—student-based peer-to-peer educator interventions developed to address HIV and sexual risk behaviour—across universities do not have the capacity or resources to reach a widespread population of students. This means that preventative and awareness messages only reach a smaller number of students (Campbell & MacPhail 2002). In a separate study conducted by (Peltzer & Promtussananon 2005), it was found that living away from home and a lack of parental supervision influenced students’ sexual risk behaviour. Maharaj and Cleland (2011) argued that economic disadvantages and poverty that lead to transactional sex impact students’ risk-taking practices.

At the University of the Western Cape, Abels and Blignaut (2011) found that some female students were forced by their male partners into sexual intercourse, thereby making females vulnerable to HIV infection. At the University of KwaZulu-Natal, Mutinta *et al.* (2013) argued that the process of striving for the long-term goals of marriage influenced students’ risk taking. For example, students indicated that they needed to ‘experience’ a partner before marrying her or him. This further exposes students to multiple sexual partnerships and possibly ‘experiencing’ a partner without the use of a condom (which are both drivers of HIV). Moreover, in other research, students did not perceive themselves to be at risk by indicating that HIV was a problem for people outside the university and expressing fatigue in talking about and addressing HIV.

Other enquiries found that students lacked the experience of making risk-aware decisions regarding sexual liaisons (Palen, Smith, Flisher, Caldwell & Mpofu 2006). The current study contributes to this growing body of literature by exploring the sexual behaviour of young African students in a metropolitan university of technology in KZN to understand the social factors underlying their risk of HIV infection

## Locating the Kwazulu-Natal epidemic

The HIV epidemic is unevenly distributed in South Africa. KZN Province is a pivot of the HIV epidemic in South Africa, with HIV prevalence at 17.8% (Mutinta *et al.* 2012; UNAIDS 2013). This province has the second largest provincial population (10.8 million) and is one of the poorest provinces in the country (www.statssa.gov.za). In KZN, prevalence was 6.1% in students overall and 8.7% in African Black students, when compared to 0.5% of the Indian population in a survey of 22 institutions of higher learning in 2008–2009 (HEAIDS 2010; Mantell, Smit, Exner, Mabude, Hoffman, Beksinska, *et al*. 2015). Despite HIV/AIDS interventions targeted at university students, sexual practices that place this group at risk of HIV infection, other STIs, as well as unintended pregnancy continue to be a serious health concern (Madumo *et al.* 2015; Mutinta *et al.* 2013). However, there is a lack of studies on the reasons why students engage in sexual risk behaviour.

The study described here was undertaken as part of a multi-phased research project aimed at providing a sero-prevalence baseline and an analysis of risk-taking behaviour at DUT in the eThekwini Metropolitan Municipality area. In 2012, the estimated HIV prevalence was 15% in the eThekwini Metro, the largest of any metropolitan municipality in South Africa. Thus, this study further contributes to the growing literature on sexual risk-taking behaviour among young people, and students in particular, within the eThekwini Metropolitan area.

### Methods

A qualitative methodology was used to investigate individual factors that influence students’ sexual risk behaviour. In-depth interviews and focus group discussions (FGDs) were used to question students on their sexual risk behaviour in order to provide insight into the individual factors that influence sexual risk-taking behaviour among university students.

The FGDs were approximately 1 hour and 30 minutes each, conducted in English, recorded, and transcribed. FGDs use interactions between researchers and participants to generate data. Recruitment was done through the university’s student portal—an online institutional system for communicating to students. Peer educators volunteering in the university’s HIV and AIDS programme also assisted in recruiting potential respondents. In addition, detailed posters with times and venues for each focus group were posted around all of the three target campuses. Different timeslots were provided, and respondents were given carte blanche to select the most suitable timeslot for participating in the focus group. No remuneration was offered for participation.

The university under study was formed in 2002 as the result of a merger of its former technical colleges. It enrols about 23,000 students annually, has seven campuses, and offers a range of undergraduate and postgraduate programmes from six faculties: Arts, Management Sciences, Applied Sciences, Health Sciences, Accounting and Informatics, and Engineering and Built Environment. Moreover, the university offers qualifications ranging from National Diplomas, Bachelors, Masters and Doctoral degrees in technology. The university is multicultural, comprising African, Indian, White, and Coloured students, with a majority of students coming from KZN. Some students live in residences provided by the university, while others live in rented accommodation outside the university, and some live at their respective homes and commute to campus.

Three campuses within the university were selected purposively because of their close proximity to each other and due to financial and technical constraints. The research consisted of a small, exploratory study consisting of 26 African students (18 females and 8 males) with an age range between 18 and 26 years. The 26 participants represented all three of the selected campuses. African students formed the majority (78%) of the student demographic profile in the university under study. Participation in this study was voluntary; however, only 26 African students showed interest in participating. Perhaps because HIV among students remains associated with race and ‘othering’, it was very hard to recruit students from other racial demographics (Mutinta *et al.* 2012). Empirical studies have found that racial minorities are less willing than non-minority individuals to participate in health-related research (Shavers, Lynch & Burmeister 2002). Moreover, in South Africa, HIV-related stigma is layered upon other stigmas associated with race (Mbonu, van den Borne & De Vries 2009); thus, it is not surprising that only African students showed interest in participating in the study. The participation of only African students has therefore influenced the focus of this paper. Likewise, a number of students refused to be part of the study, despite being assured of confidentiality, stating that they did not have time or knowledge about the topic.

### Contrivance and ethical consideration

In total, four FGDs were conducted with a mixed-gender group of students in each. The focus groups allowed students to freely discuss their experiences and share their beliefs, perspectives, and feelings. All standard ethical procedures were followed, with particular sensitivity to issues of confidentiality and anonymity, given the focus on sexual behaviour and the relation with HIV. Ethical clearance was obtained through the DUT research ethics committee. All participants were provided with information sheets detailing the aims of the research and the research process. These information sheets were provided to the participants directly. All participants were given the opportunity to ask questions about the research, and were made aware that they could withdraw from the research at any time without negative consequences. In addition, written consent was obtained from the participants before commencement of the data collection.

While no student identified as either gay or lesbian, the nature of the focus group created an environment where students could freely declare their sexuality. This method also limited the researchers from imposing their own ideas over the students. Furthermore, focus groups helped the researchers to understand the type of discussions and language used by university students around sex and HIV.

The study first collected basic demographic information including respondents’ age, marital status, and place of residence and then the focus groups were used to probe sexual experiences and behaviour; investigating the students’ knowledge and influences of such experiences and behaviour. The respondents were also asked questions that probe the topics of sexual relationships, knowledge of transactional sex, condom use, and transmission of HIV and any experience of substance use.

### Sample characteristics

The sample consisted of 26 students (18 females and 8 males) undergraduates in the age group of 18–26 years. Previous enquiries have argued that men are often reluctant to seek access to health services and participate in health-related research (Galdas, Cheater & Marshall 2005). This perhaps explains the shortage of male participants in this study. All the participants had at least one sexual partner in their lifetime and were all sexually active at the time of data collection. Participants either stayed at a campus residence or at home. All respondents had never been married and reported a Christian affiliation.

## Findings

In line with other research (Maharaj & Cleland 2011), the respondents acknowledged the health consequences, and in particular, the transmission of HIV, related to risky sexual behaviour; however, they still reported engaging in such practices. Participants’ reasoning for engaging in sexual risky behaviour is classified into five themes: 1. The influence and pressure from peers; 2. Lack of communication between students and their parents; 3. The gendered nature of information; 4. The shortcoming of abstinence programmes; and 5. Desire for love, material gain, power, and social status. The overall theme in participants’ account of risky behaviour is that of succumbing to sustained pressure from their peers. These factors interact to influence sexual risk-taking behaviour among the participants. The essence of the overarching theme is captured in a quotation by one of the participants, who used the term ‘Very, very pressured’ to describe the narrative of students under study.

Very, very pressured. Especially in our young age, people make you feel like you are abnormal if you are not having sex, for some reason. I think maybe it is because society is expecting us to have sex … but the pressure is just too much. (R23, female)

### The influence and pressure from peers

Peer influence was cited by all the students as the most important factor that influenced students’ sexual risk-taking behaviour. The respondents suggested that their behaviour was a means of gaining entry and certification into a social peer group. Part of being accepted by peers was to emulate their behaviour; in this instance, sexual behaviour was perceived as an official group membership ‘stamp’.

The greater influence is friends because it’s like when your friends tell you I have done that, you also want to try it. We trust our friends so much in things that we shouldn’t even trust them with. (R2, female)

There was consensus among the interviewees that seeking advice from their peers regarding sexual behaviour was easier than going to an adult. They found it stress-free to converse confidently with friends of the same age group. The respondents highlighted culture and religion as a barrier to talking about sex and sexuality with older health practitioners and older community members. They further expressed fear of being judged by adults when seeking health advice, therefore relied on friends for such. This is reflected in what one of the respondents mentioned:

[we] rely on [friends]. I don’t know whether it is stigma or the fear to go to the clinic and ask for such information, because [we] can just go and ask the nurse. (R26, male)

Generally, participants highlighted that their African culture does not encourage young people to speak openly about sex with their older counterparts. Likewise, their Christian religion was against premarital sex. As a consequence, students did not open up about their sexual behaviour because of fear of being labelled as social deviants. Instead, peer reliance and peer advice were considered as the most preferred options. Similarly, the respondents reported replicating their peers’ sexual behaviours rather than following their parents’ prescriptions. The earlier participant continued:

We feel like you cannot just ask anybody about condoms because that person may feel like ‘Oh this person is thinking of getting sexually involved’ and I don’t know how to explain this, but there is this fear of being judged. (R26, male)

Perceived judgements from older counterparts, whether these judgements actually surface or not, dissuaded students from seeking advice from more matured and experienced personnel. In this regard, friends and university peers offered a safer and alternative information hub. What is more, the extract above suggests a level of self-stigma that students attached to their sexual behaviour. As a consequence, they gravitated towards peers who affirmed and accepted their behaviour.

According to the respondents, it was very rare for a student to have never engaged in any form of sexual activity. Moreover, one of the respondents mentioned that over half of all his peers had engaged in a sexual activity of some sort, and they continued to do so. He explained that students are exposed to many different types of sexual practices:

I think when you refer to sexually active, maybe you will include things like kissing, masturbation and stuff, so I think a high percentage [of my friends] are involved in sexual activities or are sexually active, depending on what course of sexual activity they are involved in. but … very few … are not exposed to those things. (R1, male)

Male participants reported that out of fear of being ostracized from a peer group, they were often pressured into earlier and frequent sexual practices with multiple sexual partners. They were labelled as ‘lame’, ‘foolish’, or isishimane (a man who does not have sex) if they did not adhere to these practices. The above respondent explained negative sentiments from peers by stating:

Peer pressure is [rough] especially for guys, if you are not sexually active, they think you are lame and you are a fool. (R1, male)

The language male students used to label each other suggested a certain level of status students attached to sexual activity, or lack thereof. Words like isishimane were used in this context derogatorily to belittle and discredit those male students who did not fit the criteria or meet the standard of the sexually active category. Language on sexual activity for male students was therefore used to negotiate, mask, affirm, or resist peer group membership.

Likewise, because of a belief that it was important to be in a sexual relationship, female students were pressured into sexual activity as a way of certifying their ‘womanhood’. As these young women desired to ‘fit-in’, gain social respectability and status from other female peers, they were expected to fulfil their femininity by engaging in sexual activity, which in turn secured their place within a female peer group. One female respondent explained:

Most of us … believe it is vital that you must have a boyfriend … We do not talk about these things with our parents and we hear of them from our friends. So, we find ourselves in relationships at a [vulnerable] age, so by the time we reach a certain age, we find that we already have an experience of what it is to be in a relationship. (R2, female)

The shared belief that it was vital to have a sexual relationship is consistent with other research which suggests that young people make an effort to consolidate information on the sexual behaviour of others and their individual sexual practices (Gebhardt, Kuyper & Greunsven 2003). Likewise, students in this study suggested that they engaged in casual sexual relationships to please others; indicating an urgency and pressure to reach sexual maturity. These findings indicate that, in the face of peer pressure, university students lack the confidence to assert risk-averse practices.

### Lack of communication between students and their parents

According to respondents and tied to peer influences, parents often acted as barricades for them to access information and knowledge about sex, sexual health, and reproductive health. While it was acknowledged that parents today are more liberal, respondents highlighted that parents tended not to speak about sexuality; rather they discouraged their children from speaking openly about sex-related issues. The respondents attributed this ‘silence’ to religion and culture, suggesting that any discussion about sex was a taboo. As a consequence, interviewees did not look to their parents for advice on sexual and reproductive health, as one respondent mentioned:

It is difficult, our parents are not able to talk to us about these things … others are still a bit shy of talking about sex but somehow they have to reach a point where they see we are growing so they have to discuss it with us. (R26, male)

In addition, students extensively discussed negative reactions from their parents when they tried to acquire information about sex. These judgements led young people to gravitate towards peers for advice about their sexual lives. Two respondents highlighted the extent of the lack of communication between parents and students by saying:

When we seek such information, there seems to be that awkwardness or generation gap between our parents and maybe our lecturers, which makes it hard for us to access such information. So, the information is better received from peer educators. (R23, female)

… Well, I find it easy when I am talking to my friends than my parents, because my parents can be very judgmental. (R24, male)

Culture was also cited in in this regard and highlighted as playing a key role in creating barriers of communication across generations. The interviewees suggested that cultural beliefs and practices led to the stigma attached to sex. Students suggested that their ‘African culture’ made it very difficult to engage freely about sex; this is especially difficult when conversing with people who are older such as parents, lecturers, and health practitioners.

For us African people, parents are not the best source of information when it comes to HIV/AIDS [or] sex, because for most of my years I grew up knowing that I was delivered by a helicopter, so, I think they (parents) are not the best in terms of delivering information. (R8, female)

What I can say is people my age find out about these things through their peers, due to the fact that African people do not talk to their elders about all the things you have asked about. (R16, female)

Consistent with other literature on young people’s sexual behaviour (Mudhovozi, Ramarumo & Sodi 2013), these findings highlight intergeneration gaps regarding communication about sexuality as a driver of peer reliance and high-risk sexual practices among students. In the absence of adult-to-child communication, university peers become the ‘communication’ alternative. Thus, given that peer pressure is common, students are left unable to negotiate and make risk-aware decisions regarding their sexual practices when communicating with their peers.

### The gendered nature of information

There was general recognition among respondents of the gender undertones involved in information about issues around sex. In particular, how they sought and obtained information around sex, sexuality, and reproductive health varied by gender. For instance, respondents suggested that certain popular print-media channels were specifically designed to attract—and address the needs of—people of different gender and age. Students used these channels as an alternative to seeking information about sex from healthcare practitioners and parents.

Female respondents, for example, suggested that they did not even ‘touch’ media communication about sexual behaviour if the medium was perceived to be directed to men. Things like colour and images of a particular medium were associated with gender, and therefore only attracted that particular gender group. These same sentiments were shared by the male respondents.

The way we seek information does vary because you have your Cosmopolitan (a magazine) for your girls and then you have your Isolezwe (newspaper) for males. (R19, male)

Female interviewees further highlighted that they often bought women’s magazines such as ‘True-Love’, ‘Destiny’, and ‘O-Magazine’. These magazines were cited as the best medium for lifestyle and health advice, although they were said to be very expensive. Respondents felt that the necessary information they need concerning their sexuality, social standing, career decision-making, empowerment, and life skills could be found through this avenue. The female respondents suggested that their older male sexual partners assisted in buying these so-called expensive magazines.

On the other hand, male respondents suggested that for health and sex advice, they relied on specific newspapers that were easily accessible and cheaper to buy. Furthermore, the male respondents reported buying these newspapers to obtain information and contacts for places that offer services such as penis enlargements, treatment of STIs, medical circumcision, and clandestine backstreet sites for their female sexual partners to terminate unplanned pregnancies. These mediums of communication influenced and pressured students to model their content, and create a desire for the material products often advertised within. The above respondent continued:

Truthfully speaking, it is very rare to find a female reading Ilanga (newspaper), and that is where the information is. There is space for improvement in that area because, you have your soccer newspapers and you have your other newspapers around other spheres for men that can put such information but then there is still that gap there. (R19, male)

Given the generation gap in communication, and the fact that peer pressure is widespread, students seek other avenues to learn about sexual behaviour. Media platforms such as magazines and newspapers are therefore another important hub of information for students. These mediums are often recommended by peers, and include in their content images and advice that further put students at pressure to model their peers.

### The shortcoming of abstinence programmes

Promoting abstinence from sexual activity was cited as a futile endeavour and that instead, abstinence programmes facilitated the spread of HIV. The respondents suggested that they often consumed abstinence messages, but did not generally abstain from sexual practices. While it was agreed that messages about abstinence were important, the respondents suggested that students’ freedom to decide whether or not to abstain from sexual activity made it hard to be sexually inactive.

I think actively promoting abstinence, is perpetuating AIDS in a way actually because [we] listen but then as soon as you have left, if you spoke against whatever it is we wanted to do, it will not help … At this time we should not dwell much on abstinence because it is not totally feasible to promote abstinence here (university) because we want to have sex, and [we] will have sex whether or not we promote abstinence. (R23, female)

This response suggests that sexual activity was common within the institution and that abstinence was perceived as an abnormal behaviour and unpopular particularly because students engaged in sexual activity to gain social acceptability. Participants felt that promoting abstinence from sex was detrimental to HIV prevention interventions.

It is not everyone who comes to [tertiary] a virgin and leaves a virgin because as much as there are abstinence messages I don’t think it gets to us, it doesn’t make that much of an impact. (R11, female)

Indeed, the respondents emphasized that abstinence messages should go ‘hand in hand’ with other prevention messages such as condom use. Abstinence among students in institutions of higher education was stated as not happening at the expected rate primarily because of peer pressure, thus meriting the need for other programmes that speak to safer sexual behaviours.

However, respondents did report knowledge of people who abstain from any sexual activity within the institution. These so-called abstaining students were said to be driven largely by religious beliefs and cultural expectations, such as waiting until marriage to have sex. Moreover, the respondents reported that some students who abstained from penetrative sexual intercourse with a partner had found other means of sexual gratification, such as the use of sex-toys and the stimulating of one’s genitals (i.e. masturbating).

I strongly believe they are abstaining … because of their Christianity. There are also those, you find that there are certain ladies with certain morals and values that they don’t want to have sex until they are married. But they use sex toys or masturbate. (R1, male)

Students’ inability to abstain from sexual activity was further linked to peer pressure, in that they felt a desire to be accepted by friends within the university. Moreover, within the context of intergenerational gap in communication, the respondents highlighted that their friends’ recommendations about sexual behaviour were to be followed. Therefore, there were very few incentives for students to abstain from sexual activity.

### Desire for love, material gain, power, and social status

All the respondents reported that sexual pressure was high for both male and female students. Among female students, the desire for material possessions and financial security ‘pushed’ them into high-risk sexual relationships. For example, females reported accommodating unfaithful sexual partners in exchange for material possessions. For female students, a social bond with a partner impacted on their sexual decision-making as it gave them social status among their peers. Respondents also mentioned female students who occasionally engaged in commercial sex work or sexual relationships with older men in order to have financial security to buy certain materials like expensive hair pieces. This was reported to be more prominent among female students’ who lived in campus residences.

… There is pressure to a higher extent … [we] see other friends having those glamorous nice cloths and will then want those things and will find like a man who is going to provide for those things. Obviously a man won’t just provide for nothing they will want something in return. If the man asks for sex we give in as well because … if you don’t give in where then will we get these glamorous things. (R2, female)

Not that the sugar daddies will force me to have sex with them but I may feel pressured so that I can keep the standard of my friends. The sugar daddies can provide me with anything all I have to do is sleep with him. (R6, female)

The degree to which transactional sex is acknowledged and spoken about from the responses above suggests that the concept of exchanging sex for material gain is common among students, and female students in particular. Moreover, one of the female students suggested that she was in a sexual relationship with more than one ‘sugar daddy’. These sugar daddies were cited as a source of financial security and material provision. Patriarchal beliefs portrayed older men as exercising their masculine obligation of providing for younger females, albeit in exchange for sexual gratification.

Male students, although younger than their sugar daddy comparators, were also pressured into exercising their masculinity through the provision of gifts. Sex was further cited as a masculine requirement. As a consequence, sexual violence among students was said to be a common occurrence in cases where sex was denied to the male partner. Sexual violence was reported to be particularly prominent among students who lived in university residences, far from the security of home and family.

The respondents suggested that peer pressure often pushed male students into forcing their female sexual partners into sexual activity. Sexual violence was later blamed on female students who could not stand their ground and firmly oppose male sexual advances. Male students read (or misread) female students bodily gestures and drew conclusions on whether or not a female wanted to have sex. This is captured by a male respondent who said:

… Sometimes they are forced into sexual activities due to the economic factors and social stuff, like sex, sometimes ladies cannot stand their ground, if they say no and they are smiling a guy will say well she is a woman and she is smiling and he will try hard to convince her, which could be another form of forcing her to have sex. So, ladies should learn to stand their ground and say it is up to me. That will stop them from being forced into sexual activities. (R7, male)

Lastly, respondents suggested that the desire for love or the fear of losing a partner often leads students into unwanted and frequent sexual activity. Again, peer pressure played a critical role in fuelling the need for emotional security and love among students. Having a sexual partner was cited as an important rite into a peer social circle and adulthood. Therefore, the fear of rejection by a sexual partner led students into risk-taking sexual activity, as one respondent noted:

I would say some people do sex just to keep their partners especially the girls, they want to keep their boyfriends and they would see that if they do not do those things my boyfriend will go to someone who will give him everything. (R14, male)

Responses such as this one suggest that students perceive sex as something that could strengthen a relationship. The idea that sex and love were mutually inclusive resonated with the students. Likewise, sex was cited as a means to pleasing a partner. Therefore, other than love and pleasure, students suggested that they engaged in sexual activity to keep their partners. This was more so for female students, who feared losing their partners to other more willing female counterparts.

## Discussion

This study has explored selected social factors that influence sexual risk-taking behaviour among African students at Durban university of Technology in KZN. The greatest influences of sexual risk-taking included interpersonal processes like succumbing to peer-norms, intergenerational gaps regarding beliefs about sexual behaviour, the socio-economic context of students, the interplay between desiring love, material gain, power, and social status, HIV messaging and programmes, and the gendered nature of sex information. These findings highlight the gap between reported HIV awareness and safer sex practices among a group of young and educated South African university students (Shefer, Clowes & Vergnani 2012; Stoebenau, Nixon, Rubincam, Willan, Zembe, Tsikoane, *et al*. 2011).

While respondents at the university are exposed to messages about sexual abstinence, this study found that such messages posed a challenge since being sexually active helped students to gain entry into a peer group. Likewise, these messages are seldom consumed by the students, and as one female respondent (R11) pointed, there exists an enduring challenge of entering and exiting the university as a virgin. Therefore, students shun HIV and STI prevention messages that solely focused on abstinence. Universities need to invest more on educating students about safer sexual practices rather than just on abstinence efforts. These safer sex interventions must also speak to individual student’s social and economic contextual needs.

Students reported a desire to belong to a social group. In these groups, sexual activity was regarded as a point of entry, thus making it hard for students to negotiate sexual abstinence. Other enquiries have found that students’ newly found freedom away from home and parental control influenced their decision to be sexually active. This study adds to this growing literature by highlighting peer pressure as a dominant factor influencing risky sexual decision-making among university students (Eaton *et al.* 2003). Findings from this study are further consistent with other international research among high school adolescents in highlighting peer pressure as a driver of risky sexual practices (Cherie & Berhane 2012). Belonging to, and depending on a peer social group was cited as an important part of being a student. Therefore, to avoid being ostracized, many students conformed to pressure and engaged in high-risk sex (Selikow, Ahmed, Flisher, Mathews & Mukoma 2009). This dependence on the peer group suggests that there is a lack of support structures for young people, and students in particular. As such, being part of the group feels essential.

Other research has argued that sexual relationships often occur within the context of material exchange whereby gifts and financial support were characterized as an important basis of a relationship (Pellitor, MacPhail, Anderson & Maman 2012; Selikow *et al.* 2009). This study reinforces the argument, and further found that sexual relationships also occurred in tandem with socially ascribed masculine and feminine roles. This occurred in the form of desiring love, material gain, power, and social status. In this context, male students gained social respectability and masculine status among their peers by providing material and financial gifts to their female sexual partners. Conversely, female students were driven into sexual relationships to gain social status among their peers by being financially and materially provided for. In this regard, female students were not particularly coerced by male partners into transactional sex. However, they were driven into it by social aspirations and other non-forced choices that lead students to sex for gain.

Consistent with other studies (Mudhovozi *et al.* 2013), it was found that students have difficulties talking about sexuality with their parents. Although the participants regarded their parents as better sources of knowledge about sex, yet they had limited access to adults, and hence relied on peers and the media for information on issues around sex and HIV. There is a need for the circulation of recent, updated, accurate, personalized, and relevant information that is easily accessible and seeks to empower both students and their parents (Gacoin 2014; James *et al.* 2004). Furthermore, information should speak to the different sexual needs of both male and female students and be packaged to attract different genders and sexual orientation. Moreover, given that there is a reliance on peers, information should empower students with skills to effectively communicate with each other about sex and risk-taking behaviour. There is also a need for culturally sensitive and religious-based programmes that could be effective in bridging the gap between parental intervention and students’ needs. The role of universities in reinforcing values and decision-making processes that can assist to help students safe from sexual risk-taking behaviour needs to be investigated further as well as trying to find innovative interventions that bridge the gap between socio-economic status, peer-pressure, and intergenerational communication gaps between parents and students.

## Conclusion

This research draws attention to the perspectives of African university students regarding their risk-taking sexual practices and factors which potentially influence such behaviour. The findings are not exhaustive in exploring contextual antecedents that shape students’ sexual practices. However, they provide an important basis in understanding key factors which expose students to HIV infections. The study highlighted peer pressure among students as an influence in promoting risky sexual behaviour. Within this context, the findings revealed that university students lack the ability to negotiate risk-aware decisions especially regarding their sexual relationships. More work is needed to bring sex and gender dialogues into the university curriculum to help address this phenomenon. Additionally, cultural and religious beliefs which are disabling intergenerational communication about sexuality need to be addressed. It is therefore important that students be provided with education on both individual and social factors that influence sexual risk behaviour that exposes them to an HIV infection. The socio-economic context of students also plays a big role and increases the vulnerability of students and puts them at risk in terms of the choices they make. University environments need to ensure that intervention programmes increase student awareness and support systems for students who may be at risk. Additionally, interplays between desiring love, material gain, power, and social status play a significant role in sexual risk-taking behaviour. Further inter-disciplinary studies need to be done to understand these dynamics and come up with interventions which can assist universities and the higher education sector to effectively support students. There is also potential work that could focus on understanding the gendered nature of sex information and sex education both of which need to be understood and unpacked for the benefit of students, parents, and university support staff responsible for developing education materials for students.

Further research needs to be done to understand the degree to which peer pressure influences sexual risk-taking behaviour amongst students in higher education institutions. From this study, we can observe that there is a need for interventions that take into account the need for addressing peer pressure as a key driver for sexual risk-taking behaviour. It is also important to add that programmes aiming at preventing sexual risk-taking behaviour need to take into account the cultural and socio-economic contexts.
